# Incidence rates of treated mental disorders before and during the COVID-19 pandemic—a nationwide study comparing trends in the period 2015 to 2021

**DOI:** 10.1186/s12888-023-05157-1

**Published:** 2023-09-13

**Authors:** Pia Jensen, Bo Engdahl, Kristin Gustavson, Ingunn Olea Lund, Johanne Hagen Pettersen, Christian Madsen, Lars Johan Hauge, Ann Kristin Skrindo Knudsen, Anne Reneflot, Ragnhild Eek Brandlistuen, Helga Ask, Ragnar Nesvåg

**Affiliations:** 1 Department of Mental Disorders, Norwegian Institute of Public Health, Oslo, Norway; 2 Department of Psychology, University of Oslo, Oslo, Norway; 3 Department of Physical Health and Ageing, Norwegian Institute of Public Health, Oslo, Norway; 4 Centre for Disease Burden, Norwegian Institute of Public Health, Bergen, Norway; 5 Department of Mental Health and Suicide, Norwegian Institute of Public Health, Oslo, Norway; 6 Department of Child Health and Development, Norwegian Institute of Public Health, Oslo, Norway

**Keywords:** Incidence rates, Mental disorders, Primary health care, Specialist health care, Health registry, COVID-19, Time trends

## Abstract

**Background:**

There is a concern that exposure to psychosocial stressors during the COVID-19 pandemic may have led to a higher incidence of mental disorders. Thus, this study aimed to compare trends in incidence rates of depressive disorder, anxiety disorders, obsessive–compulsive disorder (OCD), post-traumatic stress disorder (PTSD), and eating disorders in primary- and specialist health care before (2015–2019) and during the COVID-19 pandemic (2020–2021).

**Methods:**

We used aggregated population registry data to calculate incidence rates of mental disorders from primary- (The Norwegian Control and Payment of Health Reimbursements Registry (KUHR)) and specialist (The Norwegian Patient Registry (NPR)) health care. The analyses included all Norwegian residents aged 18–65 during the study period. Incident cases were defined as having no previous registration with the same mental disorder in KUHR (from 2006) or NPR (from 2008). We used linear prediction models and mean models to compare incidence rates and test trends before and during the pandemic.

**Results:**

During the pandemic, the incidence rates among women were higher or as predicted for OCD in specialist health care and for eating disorders in both primary- and specialist health care. These findings were strongest among women aged 18–24 years. Incidence rates for depression and phobia/OCD among both genders in primary health care and phobic anxiety disorders among both genders in specialist health care were lower or as predicted.

**Conclusion:**

The COVID-19 pandemic may have led to more women needing treatment for OCD and eating disorders in the Norwegian population. The decreased incidence rates for some disorders might indicate that some individuals either avoided seeking help or had improved mental health during the pandemic.

**Supplementary Information:**

The online version contains supplementary material available at 10.1186/s12888-023-05157-1.

## Introduction

Since the outbreak of the Coronavirus disease 2019 (COVID-19), there has been a growing concern regarding the effect of the pandemic on public mental health [1]. Governments worldwide have applied various containment measures to prevent the spread of the virus, which could negatively affect individual’s well-being and mental health [2]. Many individuals may have experienced increased stress, loneliness, and reduced social contact, which are well-known risk factors for developing mental disorders [3]. For instance, Robillard et al. [4] found an increase in the proportion of Canadian respondents without a history of mental disorders who screened positive for depression and anxiety during the pandemic. On the other hand, results from systematic reviews show that an initial increase in mental health problems during the early stages of the pandemic was followed by a decline during later stages, which indicates a high level of resilience in the population [5, 6]. Qualitative studies reveal that some individuals experienced the shift to home office and cessation of commuting created time to do more positive activities, such as spending more time with loved ones, exercising and parenting [7]. Some individuals with mental health problems reported that their life became better during the pandemic, because of a slower pace and less pressure to perform in the daily life [8]. For instance, the pandemic created a more flexible calendar, less meetings, better circadian rhythm and sleep, better family relations and more time to make decisions in everyday life. Many individuals therefore seem to either have been mostly unaffected by the pandemic or were even doing better during the pandemic [9]. This could be due to reduced risk factors and less social demands, caused by the confinement and social distancing measures. For instance, people with social anxiety may have experienced decreased stress due to social distancing measures, such as mandatory home office, homeschooling, and fewer social events.

How individuals react to challenging events is based on their personal resources. It is therefore likely that the COVID-19 pandemic impacted individuals differently based on their circumstances. For instance, younger people and people living alone have experienced more loneliness than older adults [10]. Parents may have been more affected by lockdown than adults without children, as parents have experienced increased daily hassles due to the additional stress of trying to combine home office and homeschooling [11]. Whether the pandemic has affected the relative risk of individuals being diagnosed for the first time with a mental disorder is uncertain.

Studies across several countries have investigated and compared incidence rates of specific mental disorders before and during the pandemic, but the results are inconsistent. A study from the USA used electronic health records to investigate the incidences of diagnosed eating disorders among children and young adults (0–30 years) during the first year of the pandemic [12]. Compared to 2019, the incidence of eating disorders increased in 2020, particularly among young (aged 10–19 years) females. In Lithuania, a study using data from a National health care registry found that the incidence rates for post-traumatic stress disorder (PTSD), adjustment disorder, and major depressive disorder (MDD) decreased in 2020 compared to 2018 and 2019 [13]. In line with this finding, a study from the UK also found a reduction in the incidence of primary care-recorded anxiety and depression in April 2020 compared to 2019, and the decrease was most pronounced among adults (18–64 years) [14]. By September 2020, incidence rates of anxiety and depression were similar to expected levels in England but remained lower than expected in Northern Ireland, Scotland, and Wales [14].

The previous studies have some important limitations. For instance, the incidence rate of eating disorders was only investigated among children and young adults [12], and the incidence rates of PTSD, adjustment disorder, and MDD was examined using data from an insurance registry [13]. In addition, all studies mentioned above have only used data from the first year of the pandemic [12–14].

There are many possible stressors connected to the COVID-19 pandemic, such as fear of getting infected or infecting others, grief of losing a close friend or relative, financial insecurities due to job loss or temporarily being laid off, and social isolation. Such stressors could increase mental health problems, and lead to an exacerbation or development of new stress-related disorders, such as anxiety, depression, and PTSD [15, 16]. In addition, quarantine, isolation, a sense of loss of control, and changes in routines could have a negative impact on eating behavior [17, 18], and increase symptoms or lead to the development of new eating disorders in those at risk [19].

We extend previous research by using nationwide registry data capturing all contacts with primary- and specialist health care services and examining incidence rates beyond the first year of the pandemic. Based on the above considerations, we aimed to investigate if the incidence rates of mental disorders hypothesized to be influenced by the pandemic-related stress and reduced social contact (i.e., MDD, anxiety disorders, obsessive–compulsive disorder (OCD), adjustment disorder, and eating disorders) changed during the pandemic (2020–2021) compared to pre-pandemic years (2015–2019) in primary- or specialist health care.

## Material and methods

### Material

For this registry-based study, we used data from The Norwegian Control and Payment of Health Reimbursements register (KUHR), The Norwegian Patient Registry (NPR), and The National Population Register. We received two aggregated data sets from The Norwegian Directorate of Health on the incidences of pre-selected mental diagnoses from the KUHR and the NPR separately. Patients were selected based on having no previous registration with the same mental disorder since the offset of the registry (2006 for KUHR and 2008 for NPR). The incidence data were extracted without identifying patient information other than gender and age group. All individuals in the Norwegian population aged 18–65 during the study period (2015–2021) were included in the analyses.

#### The Norwegian Control and Payment of Health Reimbursements Database (KUHR)

The KUHR contains data on all patients treated in primary health care by providing information on bills from health services reimbursed to doctors by the state. The health service system in Norway is universal and primarily publicly funded, with low out-of-pocket payments. Everyone registered as living in Norway have access to a designated General Practitioner (GP). GPs are usually the patient’s first contact with the public health care system. Based on the patients' needs, the GP may refer the patient to services in specialist health care, such as mental health hospitals or outpatient clinics. For each consultation, the GP registers one or more diagnostic codes that describe the patient’s current clinical problem. In the present study, we retrieved data on the number of incident cases of selected mental diagnoses according to the International Classification of Primary Care system, 2nd edition (ICPC-2) [20] between 2008 and 2021. Data on the following ICPC-2 diagnoses were retrieved: depressive disorder (P76), anxiety disorder (P74), phobia/OCD (P79), PTSD (P82), and anorexia nervosa/bulimia (P86; eating disorders). We only included information regarding patients’ primary diagnosis in our analyses.

#### The Norwegian Patient Registry (NPR)

The NPR contains information on all patients treated in specialist health care at a hospital, outpatient clinic, or governmentally funded private specialists. In Norway, the treatment of moderate to severe mental disorders is given by the specialist health care, and patients are usually referred by their GP. State-funded mental health care is available at a low cost for all inhabitants in the entire country. Patients pay a maximum of 3040 NOK (290 $) per year for outpatient/ambulant treatment, while inpatient treatment is free of charge. For each consultation/encounter, a health professional (medical doctor/psychiatrist/licensed psychologist) registers one or more diagnostic codes that describe the patient’s current clinical problem. In the present study, we retrieved data on the number of incident cases of selected mental disorder diagnoses between 2010 and 2021 according to the International Classification of Diseases, 10th revision (ICD-10) [21]. Data on the following ICD-10 diagnoses were retrieved: MDD (F32-F34), phobic anxiety disorders (F40), other anxiety disorders (F41), OCD (F42), adjustment disorder (F43), and eating disorders (F50). We only included information regarding patients’ primary diagnosis in our analyses.

#### The National Population Register

The National Population Register contains information on everyone that lives or has lived in Norway. In the present study, we used this register to calculate mid-year population estimates for the years 2015–2021. We calculated the mid-year population estimates for the total population (18–65 years), men and women separately, and different age groups (18–24 years, 25–39 years, and 40–56 years) within gender (see Table S1 in Supplementary Material for the mid-year population estimates).

### Methods

#### Incidence rate

Age-specific incidence rates for diagnosed mental disorders across gender were calculated separately for all included diagnostic codes for each year between 2015–2021 among individuals aged 18–65. The number of incident cases in each age group was divided by the corresponding mid-year population estimate (the denominator). In the two aggregated data sets (i.e., KUHR and NPR), individuals were defined as incident cases if they had not previously been registered with the same diagnosis in KUHR (since 2006) or NPR (since 2008). To avoid misclassification of prevalent cases as incident cases, incidence rates were calculated from 2015, allowing for at least seven years of “wash-out” period for KUHR and five years for NPR to ascertain the absence of previous registrations with the same diagnosis.

### Statistical analyses

All analyses were conducted using STATA version 17. We used linear prediction models to investigate changes in incidence rates before and during the pandemic. The predicted incidence rates in 2020 and 2021 were estimated with 95% prediction intervals (PI) based on data from the complete reference period (2015–2019). We did separate prediction models for each age group among men and women. The procedure was repeated for each diagnosis in both health registries. In cases with no linear trend in the data, predictions and 95% PI was based on the mean of the incidence rates from the reference period, and differences between pre-pandemic and pandemic years were analyzed using one-sample T-test. The significance level was set to 5% in all tests. The observed incidence rates of the included mental disorders from KUHR and NPR among men and women in 2020 and 2021 were compared with predictions based on incidence rates during the pre-pandemic years (2015–2019).

### Ethics

Approval from the Regional Committees for Medical and Health Research Ethics (REK) is not required when using aggregated and anonymous registry data in the form of summary statistics.

## Results

### Primary health care

#### Depressive disorder

**Fig.1:**
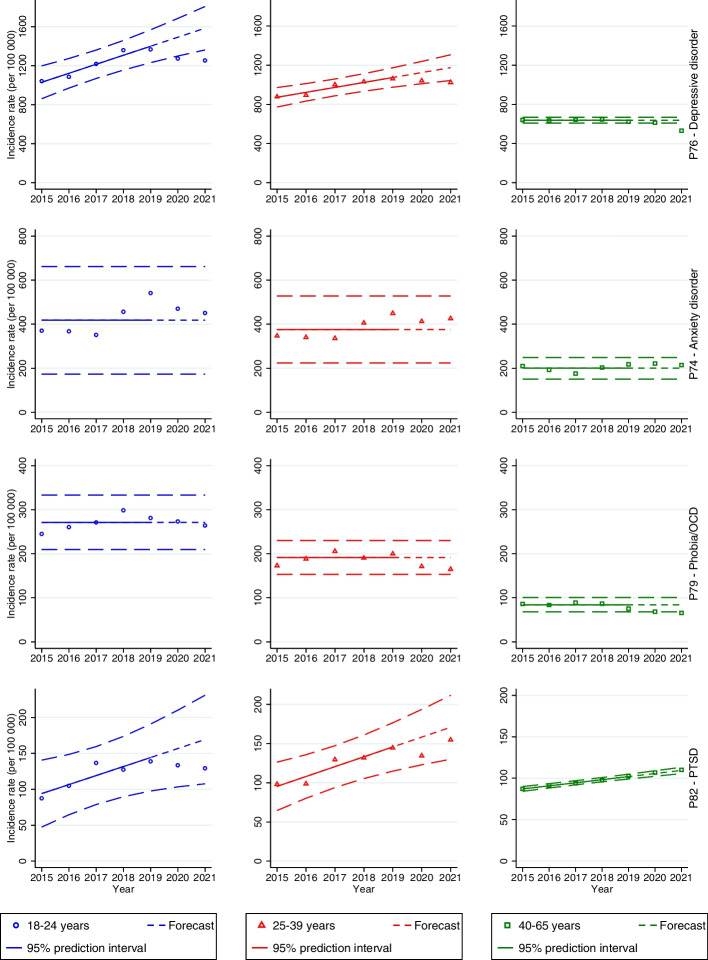
Forecasts with prediction intervals for 2020 and 2021 and observed incidence rates of primary care-recorded mental disorders among men in the period 2015–2021. When there was not a significant linear trend, the forecasts were based on the average incidence rates in the period 2015–2019

**Fig. 2:**
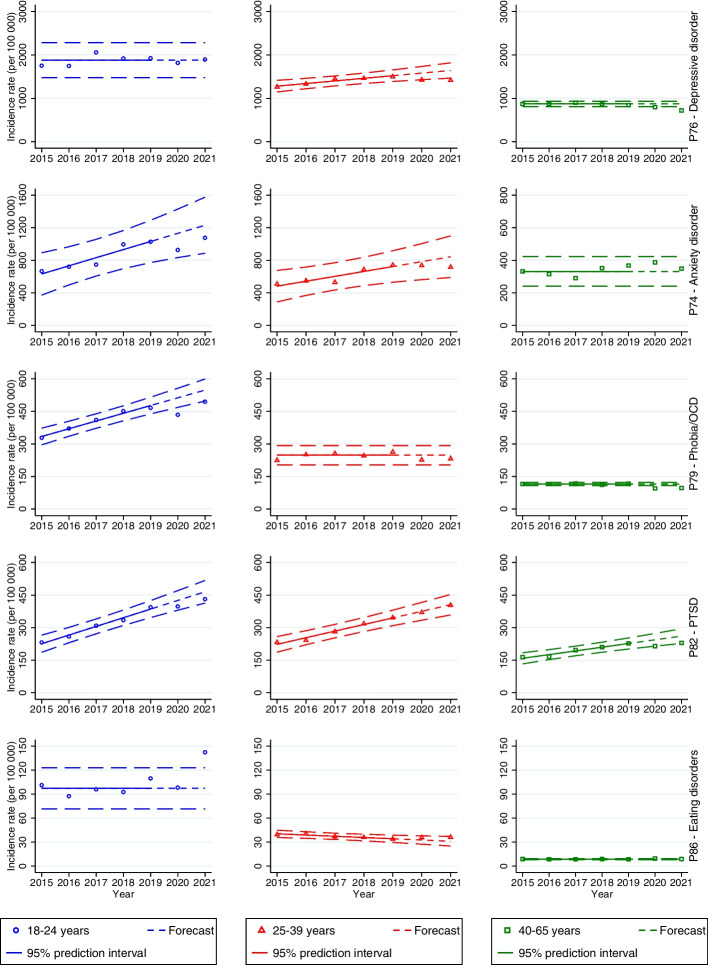
Forecasts with prediction intervals for 2020 and 2021 and observed incidence rates of primary care-recorded mental disorders among women in the period 2015–2021. When there was not a significant linear trend, the forecasts were based on the average incidence rates in the period 2015–2019

Among men, the incidence rates among the 18–24-year-olds were significantly lower than predicted during 2020 (1274 per 100,000; PI 1299–1685; *p* = 0.0367) and 2021 (1254 per 100,000; PI 1362–1807; *p* = 0.0180) (Fig. 1, first row). The incidence rate was significantly lower in 2021 among 25–39-year-olds (1025 per 100,000; PI 1044–1307; *p* = 0.0358) and 40–65-year-olds (532 per 100,000; PI 609–667; *p* = 0.0005) (Fig. 1, first row). Among women, the incidence rate was significantly lower than the pre-pandemic mean during 2020 in the age groups 25–39 years (1426 per 100,000; PI 1429–1734;* p* = 0.0472) and 40–65 years (796 per 100,000; PI 811–932; *p* = 0.0252) (Fig. 2, first row). The incidence rate was also significantly lower in 2021 in the age groups 25–39 years (1417 per 100,000; PI 1466–1818;* p* = 0.0269) and 40–65 years (721 per 100,000; PI 811–932; *p* = 0.0023).

#### Anxiety disorder

There were no significant differences between the predicted and observed incidence rates during the pandemic compared to the pre-pandemic trend (2015–2019) across both genders (see Figs. 1 and 2, second row).

#### Phobia/OCD

Among 40–65-year-old men, there was a significantly lower observed incidence rate of phobia/OCD in 2021 (65 per 100,000; PI 68–100; *p* = 0.0326) (Fig. 1, third row). Among women aged 18–24 years, the incidence rates were significantly lower than predicted during 2020 (435 per 100,000; PI 469–557; *p* = 0.0110) and 2021 (495 per 100,000; PI 497–599;* p* = 0.0438) (see Fig. 2, third row). The incidence rates were also lower than the pre-pandemic mean among women aged 40–65 in 2020 (95 per 100,000; PI 108–123; *p* = 0.0019) and 2021 (98 per 100,000; PI 108–123; *p* = 0.0030).

#### PTSD

There were no significant differences in the incidence rates among men during the pandemic compared to the pre-pandemic years (see Fig. 1, fourth row). Among women, there was a borderline significant decrease in 2020 in the age group 40–65 years (214 per 100,000; PI 215–273; *p* = 0.0491) (see Fig. 2, fourth row).

#### Eating disorders

The incidence rates of eating disorders among men in primary health care were low throughout the study period (see Table S2 in Supplementary Material). Therefore, we only present results for women. The incidence rate of eating disorders in the age group 18–24 years was significantly higher than the pre-pandemic mean in 2021 (142 per 100,000; PI 72–123; *p* = 0.0084) (see Fig. 2, fifth row). There was also a borderline significant increase in 2020 in the age group 40–65 years (10 per 100,000; PI 8–9; *p* = 0.0267).

### Specialist health care

#### Depressive disorder

**Fig.3:**
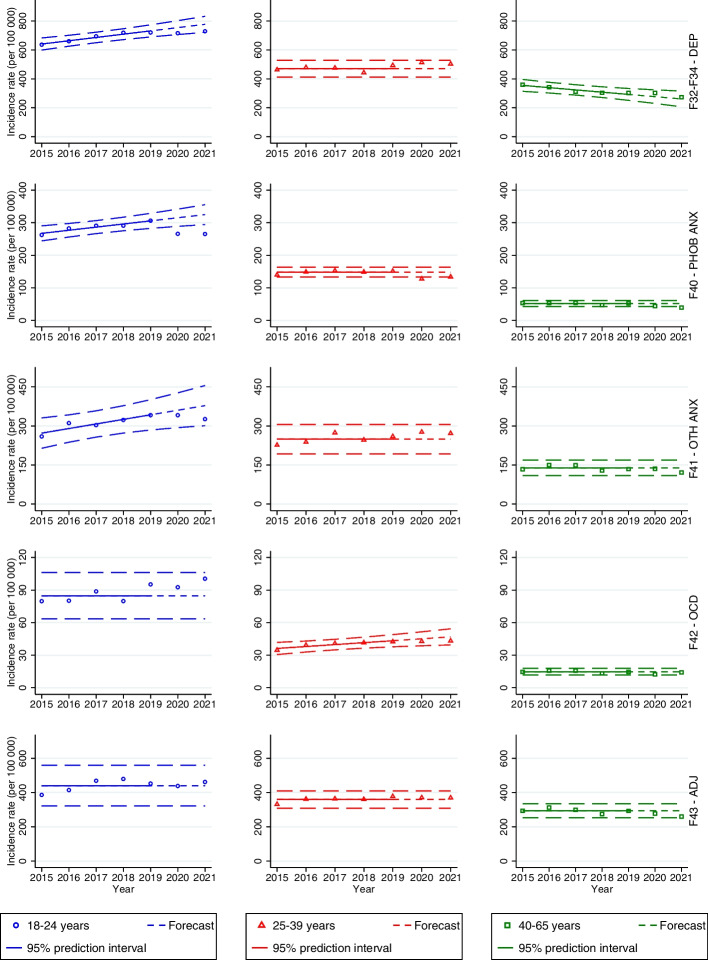
Forecasts with prediction intervals for 2020 and 2021 and observed incidence rates of specialist care-recorded mental disorders among men in the period 2015–2021. When there was not a significant linear trend, the forecasts were based on the average incidence rates in the period 2015–2019

**Fig.4:**
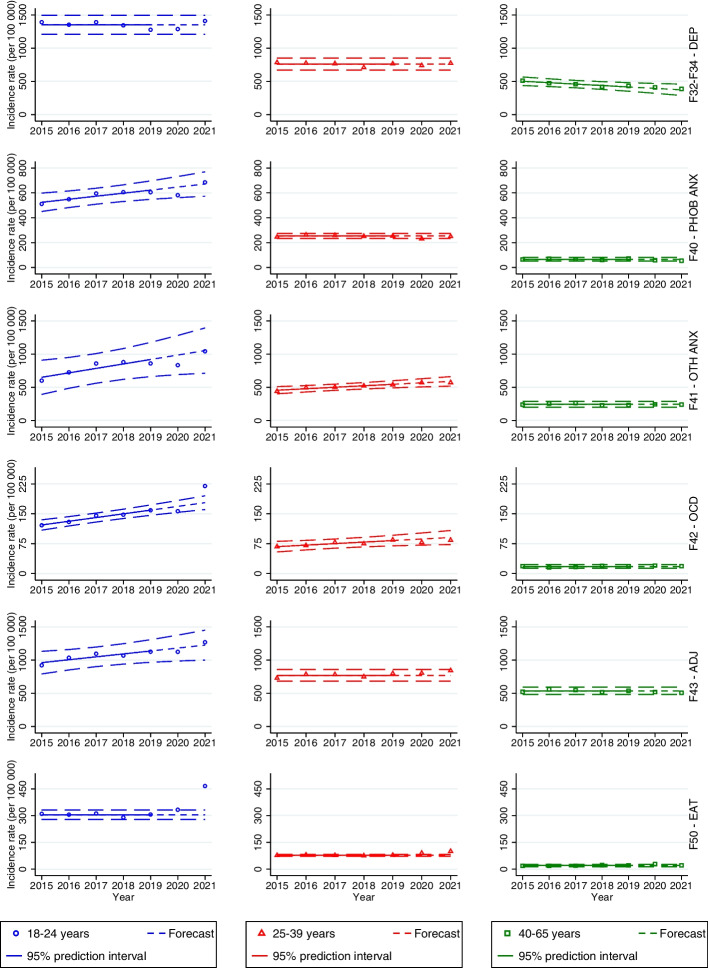
Forecasts with prediction intervals for 2020 and 2021 and observed incidence rates of specialist care-recorded mental disorders among women in the period 2015–2021. When there was not a significant linear trend, the forecasts were based on the average incidence rates in the period 2015–2019

All incidence rates of depressive disorder across both genders were within the 95% prediction intervals (see Figs. 3 and 4, first row).

#### Phobic anxiety disorders

The incidence rates among men aged 18–24 were significantly lower than predicted during 2020 (266 per 100,000; PI 289–342; *p* = 0.0095) and 2021 (265 per 100,000; PI 294–356; *p* = 0.0085) (see Fig. 3, second row). The incidence rate was also lower than predicted among men aged 25–39 in 2020 (127 per 100,000; PI 133–164; *p* = 0.0198) and men aged 40–65 in 2021 (39 per 100,000; PI 43–61; *p* = 0.0174). Among women, the incidence rate was significantly lower than predicted in the age group 25–39 in 2020 (232 per 100,000; PI 234–275; *p* = 0.0367) (see Fig. 4, second row).

#### Other anxiety disorders

There were no significant differences between the predicted and observed incidence rates of other anxiety disorders among men and women during the pandemic (see Figs. 3 and 4, third row).

#### OCD

There were no significant differences between the predicted and observed incidence rates of OCD among men (see Fig. 3, fourth row). Among women, the incidence rate was higher than predicted in 18–24-year-olds during 2021 (220 per 100,000; PI 161–195; *p* = 0.0045) (see Fig. 4, fourth row).

#### Adjustment disorders

There were no differences between the predicted and observed incidence rates of adjustment disorder during the pandemic years across both genders (see Figs. 3 and 4, fifth row).

#### Eating disorders

As with primary care-recorded eating disorders, the incidence rates of eating disorders among men in specialist health care were low throughout the study period (see Table S4 in Supplementary Material). Therefore, we only present results for women. The incidence rates among the youngest age group were significantly higher than predicted in 2020 (333 per 100,000; PI 278–331; *p* = 0.0410) and 2021 (466 per 100,000; PI 278–331; *p* = 0.0001) (see Fig. 4, sixth row). Among 25–39-year-olds, the incidence rates were higher than predicted in 2020 (91 per 100,000; PI 73–83;* p* = 0.0019) and 2021 (100 per 100,000; PI 73–83; *p* = 0.0002). The incidence rate was also higher than predicted in 2020 among 40–65-year-olds (29 per 100,000; PI 14–26; *p* = 0.0170).

### Trends and estimates of incidence rates

The yearly incidence rates and trend of each diagnosis across gender and age groups during the study period (2015–2021) are shown in Supplementary Material (Tables S2 and S4, Figures S1-S11). The predicted and observed incidence rates for mental disorders across gender and age groups during the pandemic are shown in Tables S3 and S5 in Supplementary Material.

## Discussion

Our results indicate that the COVID-19 pandemic has affected the incidence of mental disorders differently. The incidence rates of OCD in specialist health care and eating disorders in both primary- and specialist health care increased during the pandemic. The increase was only apparent among women and most pronounced in the two youngest age groups. On the other hand, in some age groups, the incidence rates of primary care-recorded depression and phobia/OCD, and specialist care-recorded phobic anxiety disorders decreased during the pandemic compared to the pre-pandemic years. There were no differences in the primary care-recorded incidence rates of anxiety disorder and PTSD and in the specialist care-recorded incidence rates of depression, other anxiety disorders, and adjustment disorders during the pandemic compared to the pre-pandemic years. The pandemic affected incidence rates of some disorders and in specific age groups, while most incidence rates followed the underlying trend from before the pandemic.

We found that primary care-recorded incidence rates of depression among men in all age groups and among women in the oldest age groups were lower than predicted. This decrease was not found in specialist health care. The result is surprising, as systematic reviews and meta-analyses have found a higher prevalence of self-reported depression in the general population during the pandemic [22–24]. However, the reduction in incidence rates are in line with both Carr et al. [14] and Kazlauskas et al. [13]. Our finding extends previous results as we also found a lower incidence rate of depression in primary health care during the second pandemic year (2021).

Further, we found decreased incidence rates of phobia/OCD among men in the oldest age group and women in the youngest and oldest age groups in KUHR, and decreased incidence rates of phobic anxiety disorders among men in all age groups and women aged 25–39 years in NPR during both pandemic years. Conversely, we found an increase in the incidence rate of specialist care-recorded OCD in 2021 among women in the youngest age group, which was not found among men. A decline in the incidence rates of phobia/OCD in primary health care and phobic anxiety disorders in specialist health care is in contrast to the prevalence literature using survey data, as some studies found elevated symptoms of phobic anxiety during quarantine [25] and lockdown [26]. However, increased incidence rates of OCD in specialist health care align with systematic reviews that have found an increased prevalence of OCD symptoms and the emergence of new symptoms [27–29].

There might be several explanations for the finding of decreased incidence rates of depression and phobia/OCD in primary health care, and phobic anxiety disorders in specialist health care during the pandemic. Firstly, the social distancing measures during the pandemic might have reduced some individuals' mental distress [13, 14]. People were encouraged, and in some periods forced, to have home office and homeschooling. Most social gatherings were canceled or reduced to a limited number of participants. Consequently, some people might have avoided situations that could trigger symptoms of mental disorders. This could have reduced the number of people receiving their first-time diagnosis of a mental disorder. Secondly, access to mental health care and GPs in Norway was reduced during periods with strict social distancing measures [30, 31]. For instance, people with newly arisen respiratory tract symptoms and those suspected infected with or exposed to the SARS-CoV-2 virus were advised not to have a face-to-face consultation with their GP or psychologist [30–33]. This might have discouraged some individuals from seeking help. Thirdly, some individuals might have feared becoming infected at the GPs office or mental health services and therefore stayed home. Since the incidence rates of depression, phobia/OCD, and phobic anxiety disorders have been either stable or had an increasing trend before the pandemic, this might indicate that some people needing mental health care did not seek help during the pandemic. If so, this is alarming as untreated mental disorders have been associated with a higher risk of suicide ideation, financial problems, family problems, and discontinuation of work and higher education [34]. Lastly, the results could be explained by true decreases or delayed help-seeking. If the results are due to delayed help-seeking, this might lead to higher incidence rates in the post-pandemic period. Future studies should investigate incidence rates across a more extended time after the pandemic to see whether the rates will rebound in the coming years.

Lastly, we found that the incidence rates of primary- and specialist care-recorded eating disorders increased considerably during the pandemic among women. The increase was apparent across all age groups, except those aged 25–39 years in KUHR, but was most prominent among the youngest age group in 2021. In primary- and specialist health care, the observed incidence rate among 18–24-year-olds in 2021 was more than 40% higher than predicted. Several countries have reported an alarming increase in the prevalence of eating disorders, especially among young people [35]. Our result aligns with Taquet et al. [12], who found increased incidence rates among children and young adults (0–30 years) in 2020. However, they found the largest increase among girls aged 10–14 and 15–19. Increased use of primary- and specialist health care for eating disorders during the pandemic have previously been documented among Norwegian children and adolescents [36]. Our finding extends previous results, as we found an increase also among adult women and during the second pandemic year (2021).

The findings of increased incidence rates of OCD and eating disorders among women during the pandemic, especially in the youngest age group, might be explained in several ways. The general finding of increased incidence rates among women could reflect that women have been more vulnerable during the pandemic [37], as systematic reviews have found more mental health problems among this group compared to men and other groups [6, 38, 39]. The finding of a larger increase in incidence rates among those aged 18–24 years aligns with studies documenting that young adults might have been more affected by changes in everyday routines, strict social distancing measures, and closures of university campuses leading to homeschooling [40]. This could explain why prevalence studies have demonstrated increased symptoms of mental distress among this group during the pandemic [41, 42]. In addition, young adulthood is a period where the emergence of psychiatric disorders usually takes place [43]. For instance, OCD is more common in adolescence and adulthood among females compared to males [44]. In addition, an increasing trend of eating disorders, especially anorexia nervosa, has been reported in individuals aged 15–19 years, and this trend is found among women and not men [45]. Taken together, our results might indicate that the pandemic and its related consequences put even more young women at risk of developing OCD and eating disorders.

Regarding the increased incidence rate of OCD in 2021 among women aged 18–24, there was much focus on disinfection, cleaning, and personal hygiene to help prevent the spread of the virus. There was also much uncertainty regarding how dangerous and contagious the virus was, which resulted in a massive fear of getting infected and infecting others. This might have triggered OCD symptoms, especially among those with contamination-related symptoms, and may have led to more people being diagnosed with OCD for the first time [29]. It is uncertain how the pandemic has affected specific subtypes of OCD, as most research has focused on contamination-related OCD symptoms [28]. Our study also shows that there has been a somewhat increasing trend in the incidence rates of OCD among women in Norway during the study period, which could partly explain the result.

Regarding eating disorders, many factors could contribute to the increased incidence rates among women during the pandemic. Firstly, almost everyone experienced disruptions in their daily routines, such as periods with closed gyms and other indoor activities, which could increase concerns regarding weight and appearance [46]. In addition, social media, either in the form of harmful eating/appearance-related content or stressful and traumatic news articles related to the pandemic, could also trigger symptoms of eating disorders [46]. In general, the COVID-19 pandemic may have acted as a global stressor that triggered the development of OCD and eating disorders among vulnerable individuals.

This study has several strengths. Firstly, we used nationwide data on incidence rates from both primary- and specialist health care, thus covering Norway's entire public health care system. Secondly, our data captured all patient encounters between 2006/2008–2021 from the entire population, thus making selection bias unlikely. Thirdly, with access to incidence rates from both 2020 and 2021, we added to and extended previous research, which only investigated incidence rates during 2020, the initial stages of the pandemic. Fourthly, we rely on diagnostic codes for mental disorders diagnosed by qualified health professionals in primary- and specialist health care. The diagnostic codes are classified according to two international classification systems (ICPC-2 and ICD-10), which ensures an international standard for diagnosing health information. One Norwegian study examined the correspondence between mental disorder diagnoses based on structured diagnostic interviews (Composite International Diagnostic Interview; CIDI), and diagnoses set in primary- (KUHR) and specialist health care (NPR), and found that diagnoses in health registries have excellent specificity because of the few false positives, and moderate sensitivity [47].

Our study also has some limitations. Firstly, we investigated incidence rates in the two health registries separately. Consequently, an individual could have received a first-time diagnosis of depression in specialist health care in 2016 but have been registered with a first-time diagnosis of depression in primary health care in 2015. This could lead to a higher number of incident cases, as the same individual may be counted as a unique case in the two separate registries. Secondly, subject-level data are available from 2006 in KUHR and from 2008 in NPR. Data during the first years will not only capture true incident cases, but also prevalent cases, as individuals might have been in contact with primary- and specialist health care before the inception of the registries. Therefore, we investigated incidence rates between 2015–2021. However, this resulted in a short reference period before the pandemic outbreak (5 years), reducing the prediction models' statistical power to detect significantly changes in incidence rates during the pandemic years. Thirdly, health professionals in primary- and specialist health care might have different diagnostic practices. In primary health care, there are no need for a referral, leading to more patients presenting with a wide range of health complaints. Hence, the diagnostic practice might be more affected by pragmatic issues, such as patients requiring sick leave and different interpretations of subjective health complaints, which might increase the number of reported cases [48]. In specialist health care, patients have been referred based on having a moderate or severe mental disorder. Due to a more selected population and fewer patients who are in contact with specialist health care, this might lead to an underrepresentation of true cases [47]. However, we have no reason to believe that the putative differences in diagnostic practices in primary- or specialist health care changed during the pandemic compared to pre-pandemic years. Fourthly, in ICPC-2, phobia and OCD are merged in the same diagnostic code (P79). This makes it impossible to interpret whether the reduced incidence rates of P79 were due to changes in one or both disorders. The finding of decreased incidence rates of phobic anxiety disorders (F40) and increased incidence rates of OCD (F42) in specialist health care using ICD-10, could indicate a corresponding decrease of phobia and increase of OCD in primary health care. Lastly, the present study did not adjust for multiple comparisons, which could increase the likelihood of making a Type I error. This may affect the interpretation of results, as some of the significant findings might have occurred by chance. However, multiple comparison correction could increase the likelihood of making a Type II error, rendering the results more challenging to interpret. By presenting all *p*-values and PIs for all tests and including the descriptive trends in Supplementary Material 1, we are transparent regarding our results. This makes it possible for the readers to assess the plausibility of our findings.

## Conclusion

Our national registry-based study of incidence rates of mental disorders between 2015–2021 indicates that the COVID-19 pandemic was a global stressor that affected the development of mental disorders differently. For most mental disorders, the incidence rates were as predicted or even decreased during the pandemic in Norway. Notably, there was an increase in specialist care-recorded OCD and a sharp increase in the incidence rates of eating disorders among women in both primary- and specialist health care. Our study might indicate that abrupt changes in everyday life and societal functions are risk factors for developing new mental disorders, which have implications for clinicians in routine-care settings and mental health services during a future crisis. The trend of more patients receiving a first-time diagnosis with OCD and eating disorders among adult women is highly relevant to mental health services and policymakers beyond the pandemic context, as these patients often need mental health care treatment. Our results may inform the planning of mental health care provision during a future crisis and guide the development of interventions to prevent the negative mental health consequences of a global stressor. Future studies should investigate whether the decreased incidence rates of depression and phobia/OCD in some age groups are due to reduced help-seeking and, therefore, untreated disorders or if the social distancing measures may have acted as protective factors.

### Supplementary Information

**Additional file 1:** **Table S1.** Mid-year population estimates in the years 2015-2021. **Table S2.** Age-specific incidence rates per 100,000 and incidence of mental disorders recorded in KUHR over time, 2015-2021. **Table S3.** Age-specific incidence rates (per 100,000) in 2020 and 2021 compared with the predicted value or mean with a 95% prediction interval from the regression models and mean models based on observational data from 2015 to 2019. **Table S4.** Age-specific incidence rates per 100,000 and incidences of mental disorders recorded in NPR over time, 2015-2021. **Table S5.** Age-specific incidence rates (per 100,000) in 2020 and 2021 compared with the predicted value or mean with a 95% prediction interval from the regression models and mean models based on observational data from 2015 to 2019. **Figure S1.** Age-specific incidence rates of primary care recorded depressive disorder, 2015-2021: men (left) and women (right). **Figure S2.** Age-specific incidence rates of primary care recorded anxiety disorder, 2015-2021: men (left) and women (right). **Figure S3.** Age-specific incidence rates of primary care recorded phobia/OCD, 2015-2021: men (left) and women (right). **Figure S4.** Age-specific incidence rates of primary care recorded PTSD, 2015-2021: men (left) and women (right). **Figure S5.** Age-specific incidence rates of primary care recorded eating disorders among women, 2015-2021. **Figure S6.** Age-specific incidence rates of specialist care-recorded depressive disorder, 2015-2021: men (left) and women (right). **Figure S7.** Age-specific incidence rates of specialist care-recorded phobic anxiety disorders, 2015-2021: men (left) and women (right). **Figure S8.** Age-specific incidence rates of specialist care-recorded other anxiety disorders, 2015-2021: men (left) and women (right). **Figure S9.** Age-specific incidence rates of specialist care-recorded obsessive-compulsive disorder, 2015-2021: men (left) and women (right). **Figure S10.** Age-specific incidence rates of specialist care-recorded adjustment disorders, 2015-2021: men (left) and women (right). **Figure S11.** Age-specific incidence rates of specialist care-recorded eating disorders among women, 2015-2021.

## Data Availability

Data generated and analyzed during this study are found in the Supplementary Material.

## Abbreviations

COVID-19: Coronavirus disease 2019
OCD: Obsessive–compulsive disorder
PTSD: Post-traumatic stress disorder
MDD: Major depressive disorder
GP: General Practitioner
KUHR: The Norwegian Control and Payment of Health Reimbursements Registry
NPR: The Norwegian Patient Registry
ICPC-2: International Classification of Primary Care system, 2nd edition
ICD-10: International Classification of Diseases, 10th revision
