# Correction: Incidence rates of treated mental disorders before and during the COVID-19 pandemic—a nationwide study comparing trends in the period 2015 to 2021

**DOI:** 10.1186/s12888-023-05367-7

**Published:** 2023-11-24

**Authors:** Pia Jensen, Bo Engdahl, Kristin Gustavson, Ingunn Olea Lund, Johanne Hagen Pettersen, Christian Madsen, Lars Johan Hauge, Ann Kristin Skrindo Knudsen, Anne Reneflot, Ragnhild Eek Brandlistuen, Helga Ask, Ragnar Nesvåg

**Affiliations:** 1 Department of Mental Disorders, Norwegian Institute of Public Health, Oslo, Norway; 2 Department of Psychology, University of Oslo, Oslo, Norway; 3 Department of Physical Health and Ageing, Norwegian Institute of Public Health, Oslo, Norway; 4 Centre for Disease Burden, Norwegian Institute of Public Health, Bergen, Norway; 5 Department of Mental Health and Suicide, Norwegian Institute of Public Health, Oslo, Norway; 6 Department of Child Health and Development, Norwegian Institute of Public Health, Oslo, Norway

**Correction: BMC Psychiatry (2023) 23:668**

10.1186/s12888-023-05157-1

Following the publication of the original article [1], the authors identified errors in the Discussion section. The updated sentence is given below, and the change has been highlighted in **bold typeface**.

The sentence currently reads:

In primary- and specialist health care, the observed incidence rate among 18–24-year-olds in 2021 was more than 140% higher than predicted.

The sentence should read:

In primary- and specialist health care, the observed incidence rate among 18–24-year-olds in 2021 was more than **40%** higher than predicted.

The original article [1] has been corrected.
