# Supplementary material for: Incidence rates of treated mental disorders before and during the COVID-19 pandemic—a nationwide study comparing trends in the period 2015 to 2021
Source: BMC Psychiatry. 2023 Sep 13;23:668. doi: 10.1186/s12888-023-05157-1 (PMC10500922; doi:10.1186/s12888-023-05157-1)
Supplement: Supplementary file 1 — Additional file 1: Table S1. Mid-year population estimates in the years 2015-2021. Table S2. Age-specific incidence rates per 100,000 and incidence of mental disorders recorded in KUHR over time, 2015-2021. Table S3. Age-specific incidence rates (per 100,000) in 2020 and 2021 compared with the predicted value or mean with a 95% prediction interval from the regression models and mean models based on observational data from 2015 to 2019. Table S4. Age-specific incidence rates per 100,000 and incidences of mental disorders recorded in NPR over time, 2015-2021. Table S5. Age-specific incidence rates (per 100,000) in 2020 and 2021 compared with the predicted value or mean with a 95% prediction interval from the regression models and mean models based on observational data from 2015 to 2019. Figure S1. Age-specific incidence rates of primary care recorded depressive disorder, 2015-2021: men (left) and women (right). Figure S2. Age-specific incidence rates of primary care recorded anxiety disorder, 2015-2021: men (left) and women (right). Figure S3. Age-specific incidence rates of primary care recorded phobia/OCD, 2015-2021: men (left) and women (right). Figure S4. Age-specific incidence rates of primary care recorded PTSD, 2015-2021: men (left) and women (right). Figure S5. Age-specific incidence rates of primary care recorded eating disorders among women, 2015-2021. Figure S6. Age-specific incidence rates of specialist care-recorded depressive disorder, 2015-2021: men (left) and women (right). Figure S7. Age-specific incidence rates of specialist care-recorded phobic anxiety disorders, 2015-2021: men (left) and women (right). Figure S8. Age-specific incidence rates of specialist care-recorded other anxiety disorders, 2015-2021: men (left) and women (right). Figure S9. Age-specific incidence rates of specialist care-recorded obsessive-compulsive disorder, 2015-2021: men (left) and women (right). Figure S10. Age-specific incidence rates of specialist care-recorded adjustme [file 12888_2023_5157_MOESM1_ESM.docx]

# Supplementary Material

## Mid-year population estimates

**Table S1.** Mid-year population estimates in the years 2015-2021

|  |  | **2015** | **2016** | **2017** | **2018** | **2019** | **2020** | **2021** |
| --- | --- | --- | --- | --- | --- | --- | --- | --- |
| Population | Age | Mid-year estimates | Mid-year estimates | Mid-year estimates | Mid-year estimates | Mid-year estimates | Mid-year estimates | Mid-year estimates |
| Men | 18-24 | 245661 | 245685.5 | 245545.5 | 245397.5 | 244538.5 | 241758 | 238799 |
|  | 25-39 | 538732 | 545112 | 550673.5 | 555441 | 560729 | 565378.5 | 569438 |
|  | 40-65 | 894004.5 | 898925.5 | 902815.5 | 906790.5 | 912082.5 | 917950 | 922589 |
| Total men | 18-65 | 1678397.5 | 1689723 | 1699034.5 | 1707629 | 1717350 | 1725086.5 | 1730826 |
| Women | 18-24 | 231130.5 | 229910.5 | 229073 | 228820.5 | 228036.5 | 225987.5 | 224022 |
|  | 25-39 | 510211.5 | 516956.5 | 523287.5 | 528817 | 534284.5 | 538361.5 | 541464.5 |
|  | 40-65 | 854401.5 | 860054 | 865210 | 870085 | 874782.5 | 879402.5 | 883828.5 |
| Total women | 18-65 | 1595743.5 | 1606921 | 1617570.5 | 1627722.5 | 1637103.5 | 1643751.5 | 1649315 |
| Total population | 18-65 | 3274141 | 3296644 | 3316605 | 3335351.5 | 3354453.5 | 3368838 | 3380141 |

## Incidence rates of mental disorders in primary health care

**Table S2.** Age-specific incidence rates per 100,000 and incidence of mental disorders recorded in KUHR over time, 2015-2021.

| **KUHR** |  |  | **2015** | **2016** | **2017** | **2018** | **2019** | **2020** | **2021** |
| --- | --- | --- | --- | --- | --- | --- | --- | --- | --- |
| Diagnosis | Sex | Age | Incidence rate* | Incidence rate* | Incidence rate* | Incidence rate* | Incidence rate* | Incidence rate* | Incidence rate* |
| P76 | Men | 18-24 | 1042 | 1086 | 1219 | 1359 | 1367 | 1274 | 1254 |
|  |  | 25-39 | 878 | 895 | 1002 | 1032 | 1062 | 1042 | 1025 |
|  |  | 40-65 | 640 | 636 | 645 | 647 | 623 | 612 | 532 |
|  | Women | 18-24 | 1752 | 1746 | 2059 | 1919 | 1925 | 1816 | 1895 |
|  |  | 25-39 | 1265 | 1331 | 1450 | 1462 | 1499 | 1426 | 1417 |
|  |  | 40-65 | 870 | 874 | 901 | 868 | 846 | 796 | 721 |
| P74 | Men | 18-24 | 370 | 368 | 351 | 456 | 541 | 470 | 451 |
|  |  | 25-39 | 347 | 341 | 336 | 406 | 450 | 413 | 426 |
|  |  | 40-65 | 209 | 193 | 176 | 203 | 218 | 221 | 214 |
|  | Women | 18-24 | 667 | 721 | 748 | 996 | 1028 | 927 | 1076 |
|  |  | 25-39 | 509 | 549 | 530 | 686 | 741 | 737 | 717 |
|  |  | 40-65 | 332 | 316 | 291 | 351 | 368 | 388 | 348 |
| P79 | Men | 18-24 | 245 | 260 | 271 | 299 | 281 | 273 | 264 |
|  |  | 25-39 | 173 | 188 | 206 | 190 | 200 | 171 | 165 |
|  |  | 40-65 | 86 | 84 | 89 | 87 | 75 | 69 | 65 |
|  | Women | 18-24 | 328 | 371 | 411 | 451 | 467 | 435 | 495 |
|  |  | 25-39 | 225 | 251 | 256 | 246 | 264 | 227 | 232 |
|  |  | 40-65 | 116 | 116 | 119 | 112 | 117 | 95 | 98 |
| P82 | Men | 18-24 | 88 | 105 | 137 | 128 | 139 | 134 | 129 |
|  |  | 25-39 | 98 | 99 | 130 | 132 | 145 | 135 | 155 |
|  |  | 40-65 | 87 | 91 | 94 | 98 | 103 | 107 | 110 |
|  | Women | 18-24 | 232 | 259 | 309 | 334 | 394 | 398 | 431 |
|  |  | 25-39 | 232 | 242 | 281 | 318 | 346 | 370 | 404 |
|  |  | 40-65 | 164 | 167 | 196 | 210 | 227 | 214 | 229 |
| P86 | Men | 18-24 | 5 | 8 | 4 | 5 | 9 | 5 | 6 |
|  |  | 25-39 | 2 | 2 | 2 | 2 | 3 | 2 | 3 |
|  |  | 40-65 | 1 | 1 | 1 | 1 | 1 | 1 | 1 |
|  | Women | 18-24 | 101 | 87 | 96 | 93 | 110 | 98 | 142 |
|  |  | 25-39 | 39 | 40 | 37 | 36 | 34 | 36 | 36 |
|  |  | 40-65 | 9 | 9 | 9 | 9 | 8 | 10 | 9 |

* The denominators for calculating incidence rates are the corresponding age-specific mid-year population estimates for men and women presented in Table S1

## Trends in incidence rates in primary health care


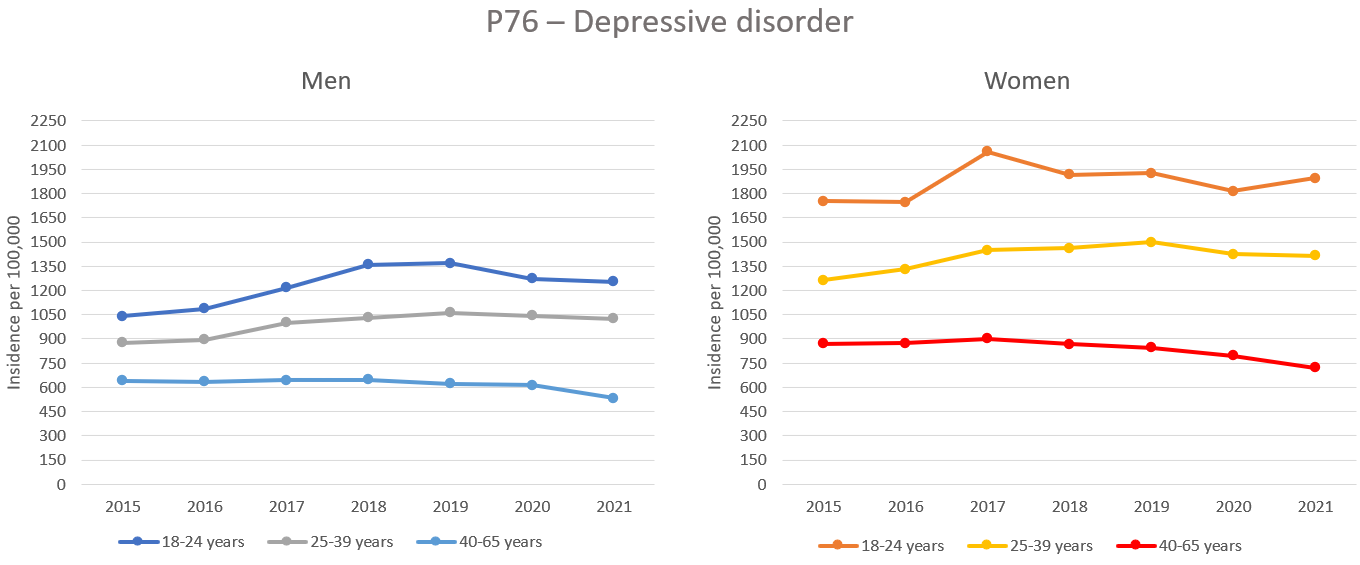


**Figure S1.** Age-specific incidence rates of primary care-recorded depressive disorder, 2015-2021: men (left) and women (right).


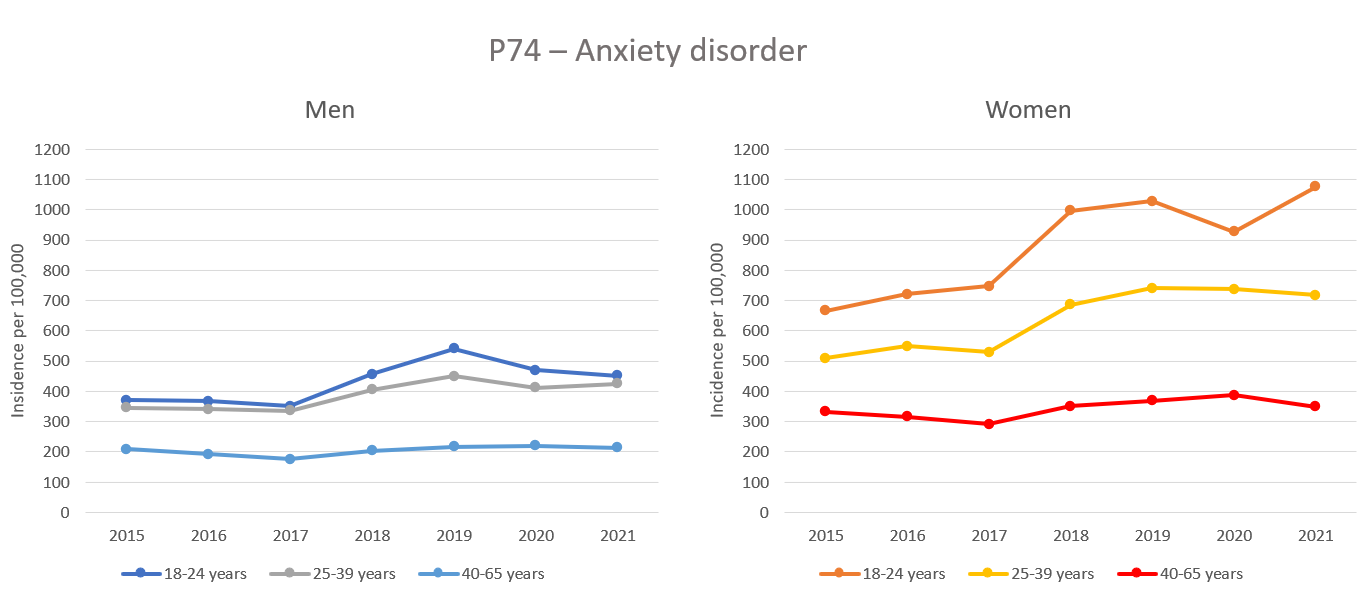


**Figure S2.** Age-specific incidence rates of primary care-recorded anxiety disorder, 2015-2021: men (left) and women (right).


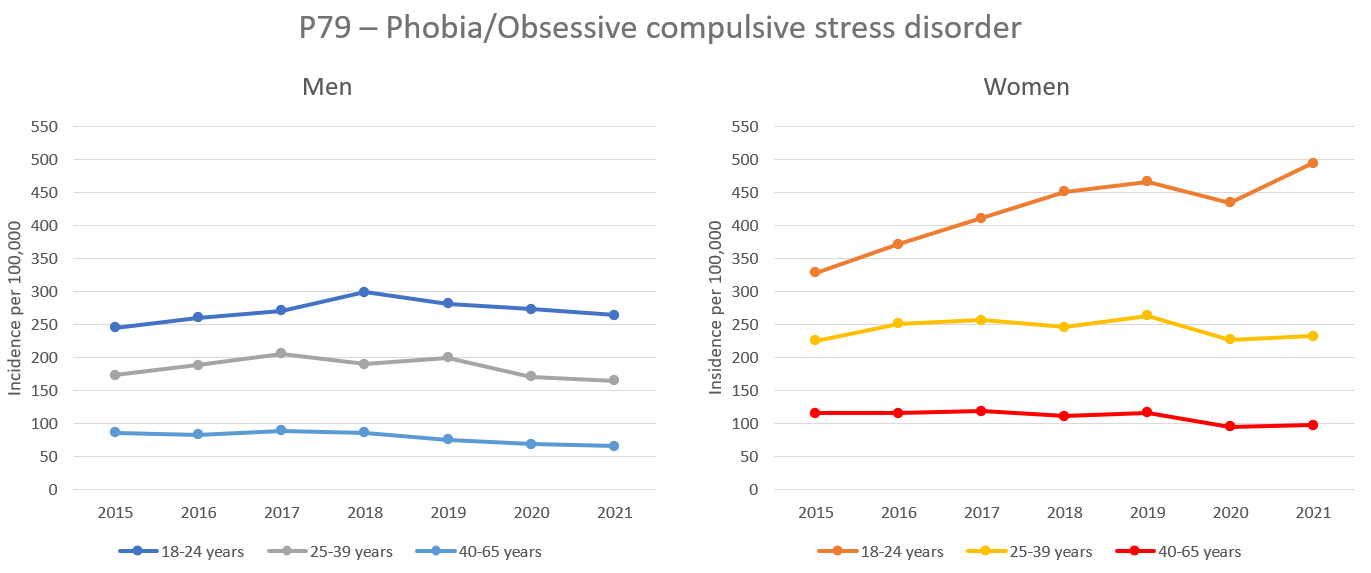


**Figure S3.** Age-specific incidence rates of primary care-recorded phobia/OCD, 2015-2021: men (left) and women (right).


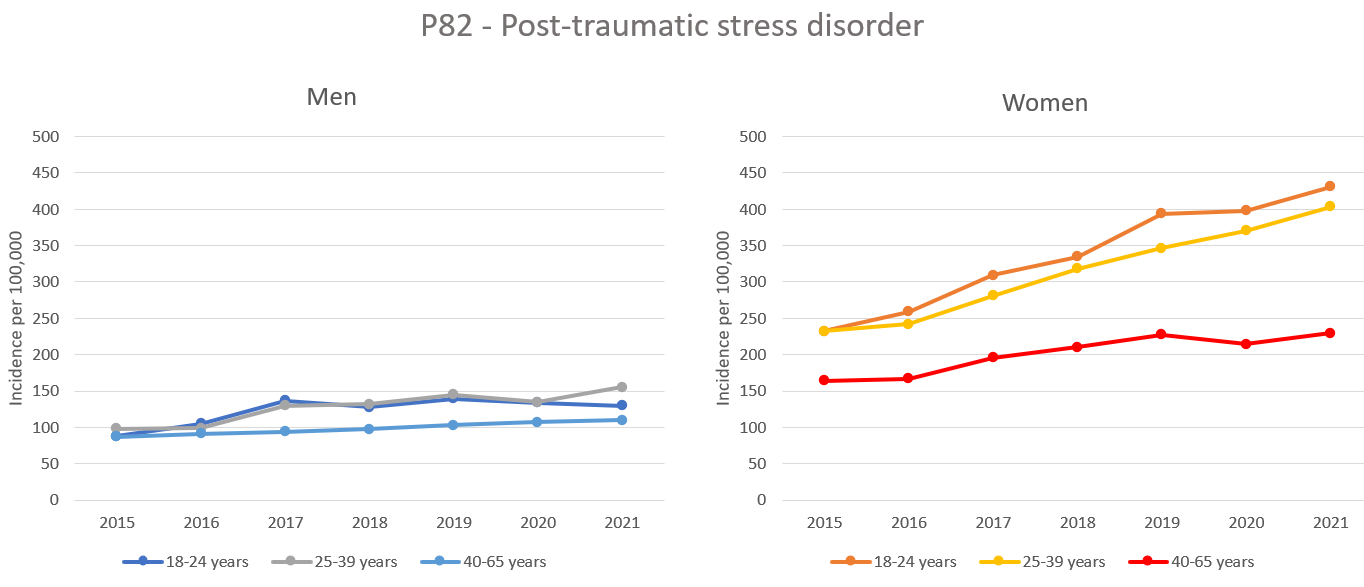


**Figure S4.** Age-specific incidence rates of primary care-recorded PTSD, 2015-2021: men (left) and women (right).


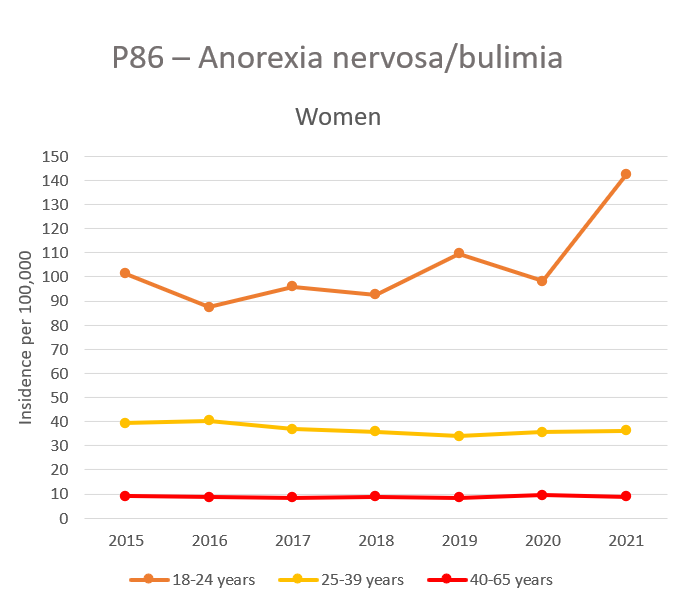


**Figure S5.** Age-specific incidence rates of primary care-recorded eating disorders among women, 2015-2021.

## Predicted and observed incidence rates of mental disorders in primary health care

**Table S3.** Age-specific incidence rates (per 100,000) in 2020 and 2021 compared with the predicted value or mean with a 95% prediction interval from the regression models and mean models based on observational data from 2015 to 2019.

|  |  |  | Incidence rate (per 100,000 mid-year population) | | | | | | | |
| --- | --- | --- | --- | --- | --- | --- | --- | --- | --- | --- |
| KUHR |  |  | 2020 | | | | 2021 | | | |
| Diagnosis | Sex | Age | Observed | Predicted (mean) | 95% PI | *p* | Observed | Predicted (mean) | 95% PI | *p* |
| P76 Depression | Men | 18-24^a^ | 1274 | 1492 | 1299-1685 | **.0367** | 1254 | 1584 | 1362-1807 | **.0180** |
|  |  | 25-39^a^ | 1042 | 1125 | 1011-1239 | .1038 | 1025 | 1176 | 1044-1307 | **.0358** |
|  |  | 40-65^b^ | 612 | 638 | 609-667 | .0683 | 532 | 638 | 609-667 | **.0005** |
|  | Women | 18-24^b^ | 1816 | 1880 | 1478-2282 | .6806 | 1895 | 1880 | 1478-2282 | .9240 |
|  |  | 25-39^a^ | 1426 | 1582 | 1429-1734 | **.0472** | 1417 | 1642 | 1466-1818 | **.0269** |
|  |  | 40-65^b^ | 796 | 872 | 811-932 | **.0252** | 721 | 872 | 811-932 | **.0023** |
| P74 Anxiety disorders | Men | 18-24^b^ | 470 | 418 | 173-662 | .5817 | 451 | 418 | 173-662 | .7235 |
|  |  | 25-39^b^ | 413 | 376 | 224-528 | .5385 | 426 | 376 | 224-528 | .4113 |
|  |  | 40-65^b^ | 221 | 200 | 150-249 | .3021 | 214 | 200 | 150-249 | .4599 |
|  | Women | 18-24^a^ | 927 | 1131 | 833-1429 | .1174 | 1076 | 1231 | 887-1575 | .2482 |
|  |  | 25-39^a^ | 737 | 784 | 562-1005 | .5511 | 717 | 844 | 588-1099 | .2136 |
|  |  | 40-65^b^ | 388 | 332 | 240-423 | .1631 | 348 | 332 | 240-423 | .6415 |
| P79 Phobia/OCD | Men | 18-24^b^ | 273 | 271 | 209-333 | .9312 | 264 | 271 | 209-333 | .7653 |
|  |  | 25-39^b^ | 171 | 192 | 153-230 | .2146 | 165 | 192 | 153-230 | .1255 |
|  |  | 40-65^b^ | 69 | 84 | 68-100 | .0570 | 65 | 84 | 68-100 | **.0326** |
|  | Women | 18-24^a^ | 435 | 513 | 469-557 | **.0110** | 495 | 548 | 497-599 | **.0438** |
|  |  | 25-39^b^ | 227 | 248 | 204-293 | .2462 | 232 | 248 | 204-293 | .3648 |
|  |  | 40-65^b^ | 95 | 116 | 108-123 | **.0019** | 98 | 116 | 108-123 | **.0030** |
| P82 PTSD | Men | 18-24^a^ | 134 | 157 | 103-210 | .2599 | 129 | 169 | 108-231 | .1309 |
|  |  | 25-39^a^ | 135 | 158 | 123-194 | .1220 | 155 | 171 | 130-212 | .2995 |
|  |  | 40-65^a^ | 107 | 106 | 102-109 | .3604 | 110 | 109 | 106-113 | .6185 |
|  | Women | 18-24^a^ | 398 | 425 | 380-470 | .1473 | 431 | 465 | 413-517 | .1291 |
|  |  | 25-39^a^ | 370 | 375 | 334-416 | .7225 | 404 | 406 | 358-453 | .8960 |
|  |  | 40-65^a^ | 214 | 244 | 215-273 | **.0491** | 229 | 261 | 227-295 | .0582 |
| P86 Eating disorders | Women | 18-24^b^ | 98 | 97 | 72-123 | .9325 | 142 | 97 | 72-123 | **.0084** |
|  |  | 25-39^a^ | 36 | 33 | 27-38 | .1615 | 36 | 31 | 25-37 | .0732 |
|  |  | 40-65^b^ | 10 | 9 | 8-9 | **.0267** | 9 | 9 | 8-9 | .3867 |

^a^Linear regression model with 95% Prediction interval (PI) based on the pre-pandemic incidence rates (2015-2019)

^b^Mean model with 95% PI based on the mean of the pre-pandemic incidence rates (2015-2019)

## Incidence rates of mental disorders in specialist health care

**Table S4.** Age-specific incidence rates per 100,000 and incidences of mental disorders recorded in NPR over time, 2015-2021.

| **NPR** |  |  | **2015** | **2016** | **2017** | **2018** | **2019** | **2020** | **2021** |
| --- | --- | --- | --- | --- | --- | --- | --- | --- | --- |
| Diagnosis | Sex | Age | Incidence rate* | Incidence rate* | Incidence rate* | Incidence rate* | Incidence rate* | Incidence rate* | Incidence rate* |
| F32-F34 | Men | 18-24 | 637 | 660 | 694 | 719 | 721 | 716 | 729 |
|  |  | 25-39 | 463 | 479 | 476 | 443 | 493 | 514 | 504 |
|  |  | 40-65 | 360 | 343 | 311 | 303 | 302 | 302 | 274 |
|  | Women | 18-24 | 1392 | 1354 | 1392 | 1345 | 1278 | 1289 | 1410 |
|  |  | 25-39 | 785 | 776 | 769 | 710 | 766 | 740 | 775 |
|  |  | 40-65 | 512 | 474 | 459 | 419 | 432 | 408 | 385 |
| F40 | Men | 18-24 | 263 | 282 | 291 | 291 | 306 | 266 | 265 |
|  |  | 25-39 | 140 | 149 | 153 | 149 | 151 | 128 | 134 |
|  |  | 40-65 | 53 | 53 | 54 | 47 | 53 | 44 | 39 |
|  | Women | 18-24 | 511 | 549 | 595 | 606 | 605 | 581 | 685 |
|  |  | 25-39 | 245 | 262 | 259 | 252 | 254 | 232 | 251 |
|  |  | 40-65 | 65 | 72 | 64 | 62 | 72 | 58 | 54 |
| F41 | Men | 18-24 | 260 | 311 | 303 | 323 | 341 | 341 | 326 |
|  |  | 25-39 | 226 | 238 | 274 | 246 | 260 | 277 | 272 |
|  |  | 40-65 | 134 | 150 | 149 | 128 | 134 | 136 | 122 |
|  | Women | 18-24 | 601 | 726 | 859 | 880 | 860 | 834 | 1043 |
|  |  | 25-39 | 442 | 495 | 505 | 523 | 540 | 572 | 574 |
|  |  | 40-65 | 241 | 257 | 262 | 230 | 233 | 244 | 240 |
| F42 | Men | 18-24 | 80 | 80 | 89 | 80 | 95 | 93 | 101 |
|  |  | 25-39 | 35 | 39 | 41 | 42 | 42 | 43 | 43 |
|  |  | 40-65 | 15 | 16 | 16 | 13 | 15 | 12 | 14 |
|  | Women | 18-24 | 121 | 130 | 145 | 148 | 159 | 157 | 220 |
|  |  | 25-39 | 68 | 70 | 78 | 75 | 85 | 78 | 84 |
|  |  | 40-65 | 19 | 15 | 17 | 19 | 18 | 20 | 19 |
| F43 | Men | 18-24 | 386 | 415 | 469 | 480 | 453 | 438 | 462 |
|  |  | 25-39 | 332 | 362 | 364 | 361 | 378 | 371 | 370 |
|  |  | 40-65 | 294 | 312 | 299 | 274 | 293 | 277 | 261 |
|  | Women | 18-24 | 922 | 1036 | 1096 | 1068 | 1126 | 1125 | 1269 |
|  |  | 25-39 | 729 | 786 | 784 | 751 | 800 | 804 | 844 |
|  |  | 40-65 | 524 | 562 | 547 | 518 | 539 | 518 | 509 |
| F50 | Men | 18-24 | 21 | 23 | 19 | 23 | 24 | 24 | 27 |
|  |  | 25-39 | 6 | 7 | 7 | 8 | 6 | 8 | 10 |
|  |  | 40-65 | 3 | 2 | 3 | 3 | 4 | 6 | 5 |
|  | Women | 18-24 | 310 | 305 | 312 | 290 | 306 | 333 | 466 |
|  |  | 25-39 | 77 | 80 | 77 | 76 | 79 | 90 | 100 |
|  |  | 40-65 | 19 | 18 | 19 | 23 | 21 | 29 | 22 |

* The denominators for calculating incident rates are the corresponding age-specific mid-year population estimates for men and women presented in Table S1

## Trends in incidence rates in specialist health care


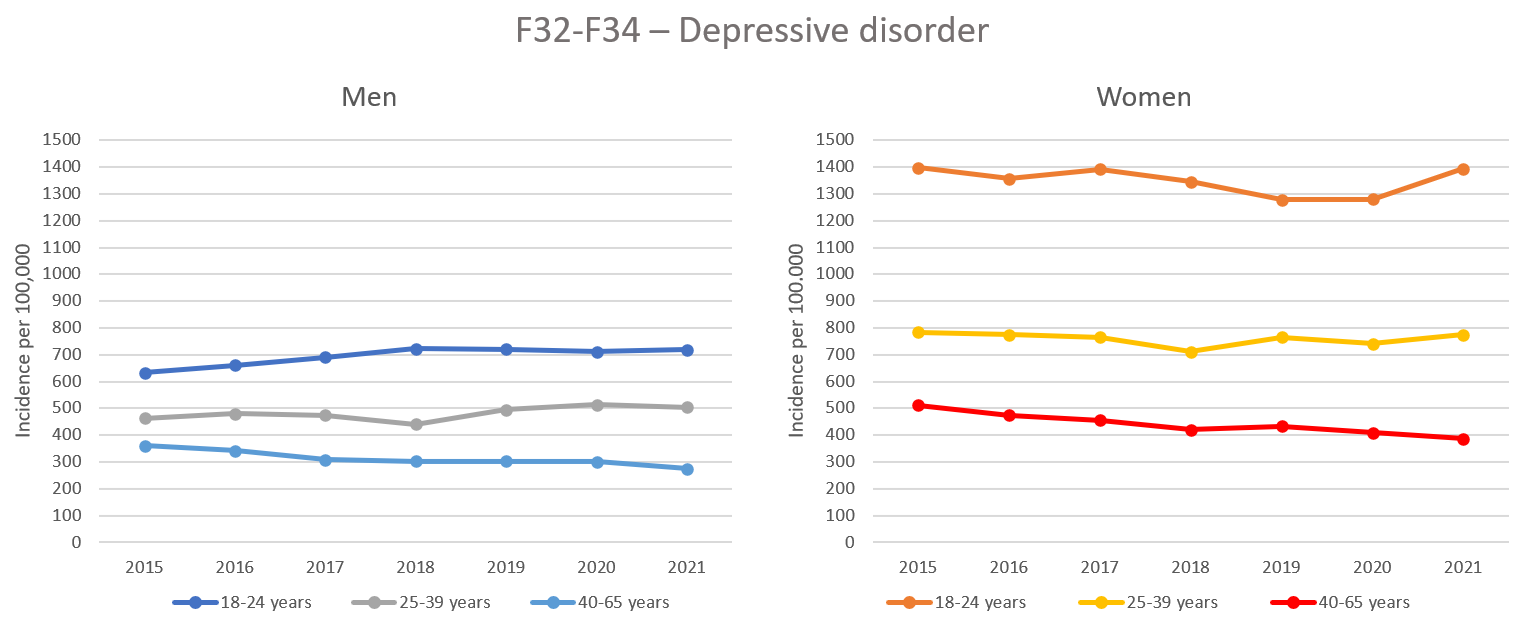


**Figure S6.** Age-specific incidence rates of specialist care-recorded depressive disorder, 2015-2021: men (left) and women (right).


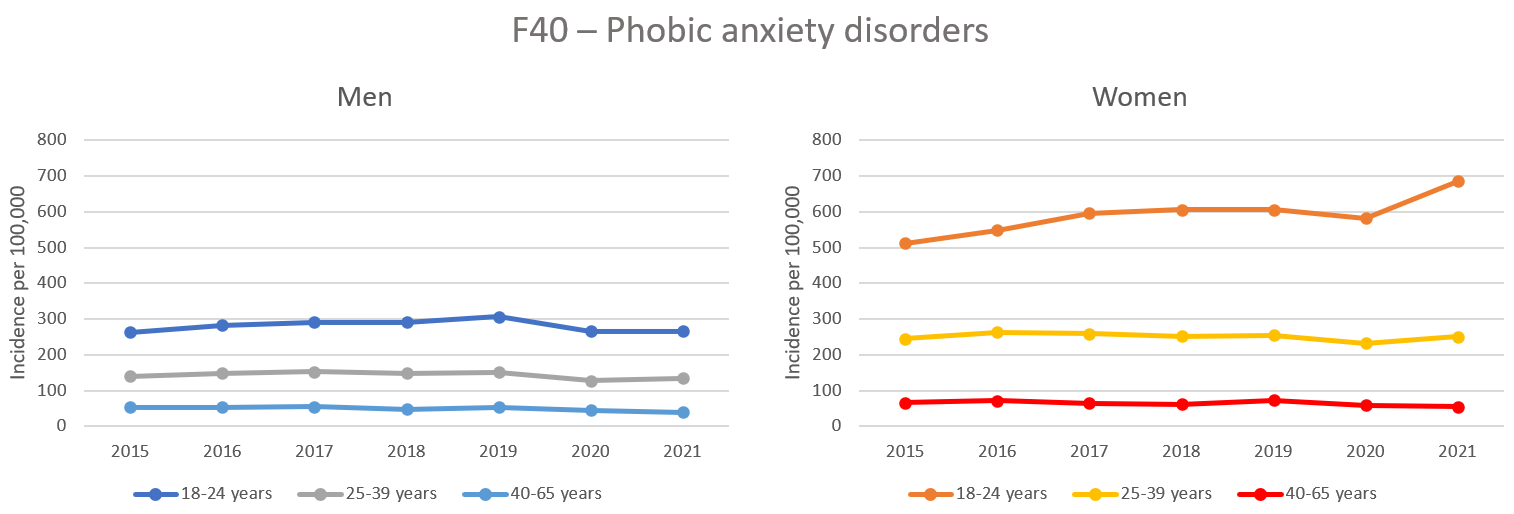


**Figure S7.** Age-specific incidence rates of specialist care-recorded phobic anxiety disorders, 2015-2021: men (left) and women (right).


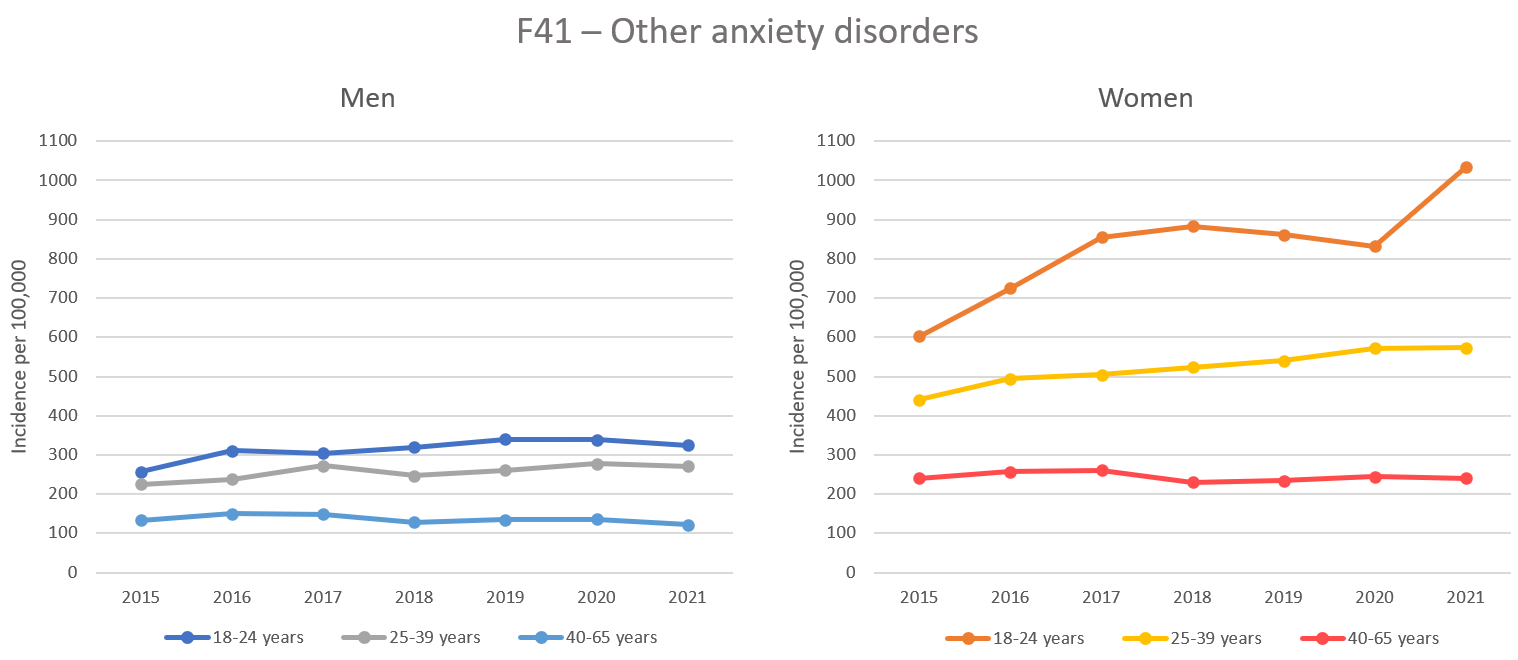


**Figure S8.** Age-specific incidence rates of specialist care-recorded other anxiety disorders, 2015-2021: men (left) and women (right).


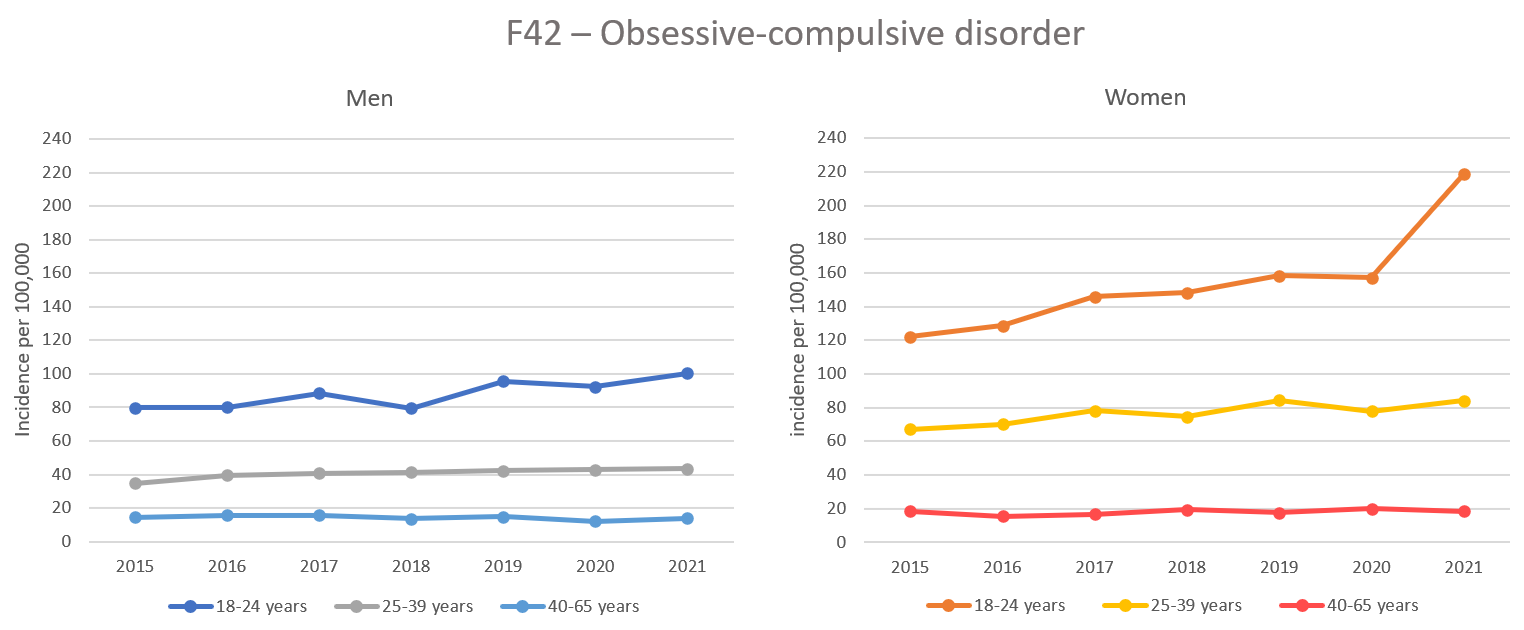


**Figure S9.** Age-specific incidence rates of specialist care-recorded obsessive-compulsive disorder, 2015-2021: men (left) and women (right).


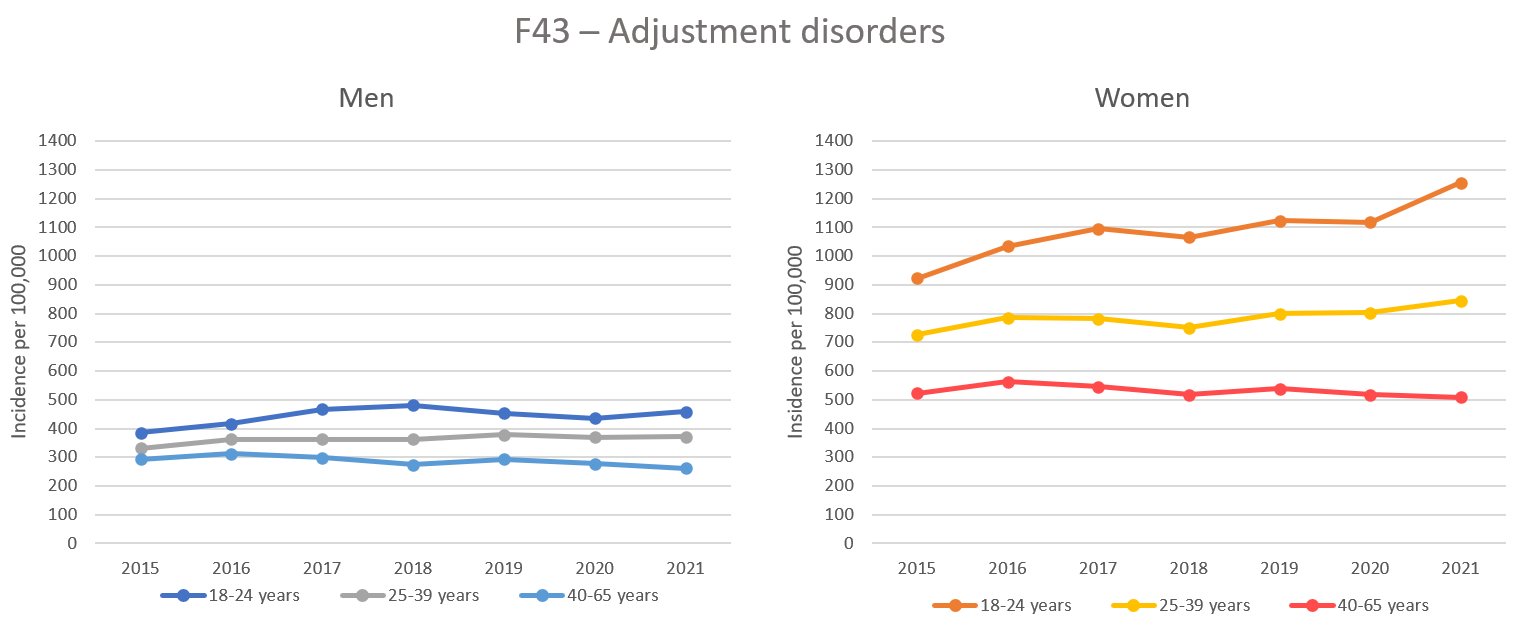


**Figure S10.** Age-specific incidence rates of specialist care-recorded adjustment disorders, 2015-2021: men (left) and women (right).


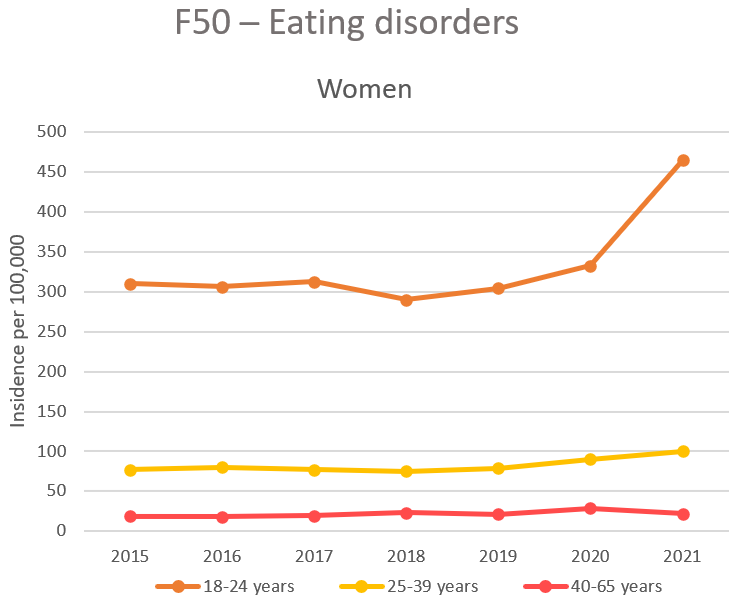


**Figure S11.** Age-specific incidence rates of specialist care-recorded eating disorders among women, 2015-2021.

## Predicted and observed incidence rates of mental disorders in specialist health care

**Table S5.** Age-specific incidence rates (per 100,000) in 2020 and 2021 compared with the predicted value or mean with a 95% prediction interval from the regression models and mean models based on observational data from 2015 to 2019.

|  |  |  | Incidence rate (per 100,000) | | | | | | | |
| --- | --- | --- | --- | --- | --- | --- | --- | --- | --- | --- |
| NPR |  |  | 2020 | | | | 2021 | | | |
| Diagnosis | Sex | Age | Observed | Predicted (mean) | 95% PI | *p* | Observed | Predicted (mean) | 95% PI | *p* |
| F32-F34 Depression | Men | 18-24^a^ | 716 | 754 | 706-902 | .0848 | 729 | 777 | 722-832 | .0706 |
|  |  | 25-39^b^ | 514 | 471 | 413-529 | .1081 | 504 | 471 | 413-529 | .1905 |
|  |  | 40-65^a^ | 302 | 277 | 230-324 | .1854 | 274 | 262 | 207-316 | .5138 |
|  | Women | 18-24^b^ | 1289 | 1352 | 1210-1494 | .2822 | 1410 | 1352 | 1210-1494 | .3215 |
|  |  | 25-39^b^ | 740 | 761 | 671-851 | .5484 | 775 | 761 | 671-851 | .7006 |
|  |  | 40-65^a^ | 408 | 395 | 321-469 | .6068 | 385 | 373 | 288-459 | .6843 |
| F40 Phobic anxiety | Men | 18-24^a^ | 266 | 315 | 289-342 | **.0095** | 265 | 325 | 294-356 | **.0085** |
|  |  | 25-39^b^ | 128 | 148 | 133-164 | **.0198** | 134 | 148 | 133-164 | .0591 |
|  |  | 40-65^b^ | 44 | 52 | 43-61 | .0687 | 39 | 52 | 43-61 | **.0174** |
|  | Women | 18-24^a^ | 581 | 647 | 562-731 | .0919 | 685 | 671 | 573-769 | .6863 |
|  |  | 25-39^b^ | 232 | 255 | 234-275 | **.0367** | 251 | 255 | 234-275 | .6303 |
|  |  | 40-65^b^ | 58 | 67 | 53-81 | .1633 | 54 | 67 | 53-81 | .0622 |
| F41 Other anxiety disorders | Men | 18-24^a^ | 341 | 360 | 294-427 | .4292 | 326 | 378 | 301-455 | .1223 |
|  |  | 25-39^b^ | 277 | 249 | 193-306 | .2446 | 272 | 249 | 193-306 | .3257 |
|  |  | 40-65^b^ | 136 | 139 | 109-169 | .7704 | 122 | 139 | 109-169 | .1739 |
|  | Women | 18-24^a^ | 834 | 986 | 691-1282 | .1989 | 1043 | 1054 | 713-1394 | .9294 |
|  |  | 25-39^a^ | 572 | 568 | 507-630 | .8678 | 574 | 591 | 519-662 | .5037 |
|  |  | 40-65^b^ | 244 | 245 | 202-288 | .9765 | 240 | 245 | 202-288 | .7789 |
| F42 OCD | Men | 18-24^b^ | 93 | 85 | 63-106 | .3630 | 101 | 85 | 63-106 | .1100 |
|  |  | 25-39^a^ | 43 | 45 | 39-52 | .3359 | 43 | 47 | 39-54 | .2116 |
|  |  | 40-65^b^ | 12 | 15 | 12-18 | .0850 | 14 | 15 | 12-18 | .5441 |
|  | Women | 18-24^a^ | 157 | 169 | 154-183 | .0849 | 220 | 178 | 161-195 | **.0045** |
|  |  | 25-39^a^ | 78 | 87 | 71-102 | .1839 | 84 | 90 | 73-108 | .3186 |
|  |  | 40-65^b^ | 20 | 18 | 13-22 | .2278 | 19 | 18 | 13-22 | .5618 |
| F43 Adjustment disorders | Men | 18-24^b^ | 438 | 441 | 322-559 | .9640 | 462 | 441 | 322-559 | .6434 |
|  |  | 25-39^b^ | 371 | 359 | 309-410 | .5779 | 370 | 359 | 309-410 | .5898 |
|  |  | 40-65^b^ | 277 | 294 | 253-336 | .3044 | 261 | 294 | 253-336 | .0848 |
|  | Women | 18-24^a^ | 1125 | 1182 | 986-1377 | .4235 | 1269 | 1225 | 1000-1451 | .5816 |
|  |  | 25-39^b^ | 804 | 770 | 681-859 | .3432 | 844 | 770 | 681-859 | .0814 |
|  |  | 40-65^b^ | 518 | 538 | 483-593 | .3817 | 509 | 538 | 483-593 | .2222 |
| F50 Eating disorders | Women | 18-24^b^ | 333 | 305 | 278-331 | **.0410** | 466 | 305 | 278-331 | **.0001** |
|  |  | 25-39^b^ | 90 | 78 | 73-83 | **.0019** | 100 | 78 | 73-83 | **.0002** |
|  |  | 40-65^b^ | 29 | 20 | 14-26 | **.0170** | 22 | 20 | 14-26 | .4596 |

^a^Linear regression model with 95% PI based on the pre-pandemic incidence rates (2015-2019)

^b^Mean model with 95% PI based on the mean of the pre-pandemic incidence rates (2015-2019)
